# Combined Effect of Bortezomib and Menadione Sodium Bisulfite on Proteasomes of Tumor Cells: The Dramatic Decrease of Bortezomib Toxicity in a Preclinical Trial

**DOI:** 10.3390/cancers10100351

**Published:** 2018-09-25

**Authors:** Tatiana M. Astakhova, Alexey V. Morozov, Pavel A. Erokhov, Maria I. Mikhailovskaya, Sergey B. Akopov, Natalia I. Chupikova, Ruslan R. Safarov, Natalia P. Sharova

**Affiliations:** 1 Laboratory of Biochemistry of Ontogenesis Processes, Koltzov Institute of Developmental Biology, Russian Academy of Sciences, 26 Vavilov Street, 119334 Moscow, Russia; tastakhova@bk.ru (T.M.A.); erokhov.p@gmail.com (P.A.E.); maria@actremed.ru (M.I.M.); chupikova@actremed.ru (N.I.C.); safarov@actremed.ru (R.R.S.); 2 Laboratory of Regulation of Intracellular Proteolysis, Engelhardt Institute of Molecular Biology, Russian Academy of Sciences, 32 Vavilov Street, 119991 Moscow, Russia; runkel@inbox.ru; 3 Laboratory of Human Genes Structure and Functions, Shemyakin–Ovchinnikov Institute of Bioorganic Chemistry of Russian Academy of Sciences, 16/10 Miklukho-Maklay Street, 117997 Moscow, Russia; sergeyakopov@mail.ru

**Keywords:** target and drug discovery, proteasome forms, bortezomib, menadione sodium bisulfite, hepatocellular carcinoma, mammary adenocarcinoma, Lewis lung carcinoma, antitumor effect in vivo, acute toxicity

## Abstract

Tumor growth is associated with elevated proteasome expression and activity. This makes proteasomes a promising target for antitumor drugs. Current antitumor drugs such as bortezomib that inhibit proteasome activity have significant side effects. The purpose of the present study was to develop effective low-toxic antitumor compositions with combined effects on proteasomes. For compositions, we used bortezomib in amounts four and ten times lower than its clinical dose, and chose menadione sodium bisulfite (MSB) as the second component. MSB is known to promote oxidation of NADH, generate superoxide radicals, and as a result damage proteasome function in cells that ensure the relevance of MSB use for the composition development. The proteasome pool was investigated by the original native gel electrophoresis method, proteasome chymotrypsin-like activity—by Suc-LLVY-AMC-hydrolysis. For the compositions, we detected 10 and 20 μM MSB doses showing stronger proteasome-suppressing and cytotoxic in cellulo effects on malignant cells than on normal ones. MSB indirectly suppressed 26S-proteasome activity in cellulo, but not in vitro. At the same time, MSB together with bortezomib displayed synergetic action on the activity of all proteasome forms in vitro as well as synergetic antitumor effects in cellulo. These findings determine the properties of the developed compositions in vivo: antitumor efficiency, higher (against hepatocellular carcinoma and mammary adenocarcinoma) or comparable to bortezomib (against Lewis lung carcinoma), and drastically reduced toxicity (LD50) relative to bortezomib. Thus, the developed compositions represent a novel generation of bortezomib-based anticancer drugs combining high efficiency, low general toxicity, and a potentially expanded range of target tumors.

## 1. Introduction

Proteasomes, multicatalytic proteases, regulate cellular processes as well as cell proteome by the production of biologically active peptides and degradation of growth factors, receptors, signal-pathway components, transcription factors and other proteins [1,2,3]. In different animal and human malignant tumors, proteasome expression and chymotrypsin-like (ChTL) activity are enhanced relative to normal tissues [4,5,6,7,8]. Implanted Walker 256 carcinosarcoma regressed after a short period of growth in Brattleboro rats with the hereditary defect of arginine-vasopressin synthesis in the hypothalamus but not in control Wag rats with normal arginine-vasopressin synthesis. The period of tumor regression coincided with the essential decrease in proteasome expression and ChTL-activity [9]. Altogether, these facts indicate that tumor growth is highly dependent on the functional activity of proteasomes. Therefore, proteasomes are a promising target for antitumor drugs.

Since 2003, bortezomib, a reversible competitive inhibitor of proteasome ChTL-activity, has been used in clinical practice as an antitumor drug [10,11]. Bortezomib is applied mainly against several hematologic malignancies, which are more sensitive to this drug than solid tumors. At the same time, bortezomib effects on solid tumors are still under investigation [12,13]. The major obstacles to bortezomib therapy are serious side effects including peripheral neuropathy, thrombocytopenia, gastrointestinal disturbances, fatigue, and perhaps cardiac abnormality [11,14,15], which can be the reason for effective dose reductions or even discontinuations.

To enhance the antitumor effects and reduce general toxicity associated with bortezomib, proteasome inhibitors of the newer generation are being developed. The modifications include irreversible proteasome inhibition, inhibition of all three enzymatic sites, and oral rather than intravenous administration [11]. Clinical studies of carfilzomib (an irreversible inhibitor of the ChTL-site) and ixazomib (an orally bioavailable proteasome inhibitor) on patients with multiple myeloma show that in some instances tumors resistant to bortezomib are sensitive to the newer agents. However, these agents also cause serious side effects [11]. Another approach to reducing side effects and general toxicity of proteasome inhibitors is the development of immune subunit inhibitors not affecting the proteolytic sites of housekeeping constitutive proteasomes [16].

We proposed an alternative approach and developed two-component antitumor compositions with a combined effect on proteasomes. The first component is bortezomib in much lower amounts compared to its current clinical dose. The second one is menadione sodium bisulfite (MSB) (Vikasolum, vitamin K3). MSB promotes oxidation of NADH, generates superoxide radicals and as a result damages proteasome function in the intracellular medium [17,18,19]. Besides, MSB can cause tumor cell death [20]. These facts ensure the relevance of MSB use for the composition development. We tested the compositions of bortezomib and MSB and proved their antitumor effects and low toxicity in a preclinical trial.

## 2. Results and Discussion

### 2.1. Cytotoxic Action of MSB

The MSB doses for antitumor compositions were determined by comparing the cytotoxic effect of MSB against malignant cells (human epidermoid carcinoma A-431, mouse hepatocellular carcinoma Hepa 1-6, and mouse colon adenocarcinoma C26) and immortalized cells (human immortalized keratinocytes HaCat) with its effect against loach embryo cells. Loach embryos developing outside the maternal body represent a unique in vivo model in general. It is a mini-model convenient for the comparative study of some processes in cell lines and organism. It is possible to expose dividing normal cells in this model as well as dividing malignant and immortalized cells of the cell lines to MSB in a similar manner, by the addition of the drug into the culture medium. Therefore, this model allows us to analyze the difference in response of the malignant cells and organism to damaging effects.

**Figure 1:**
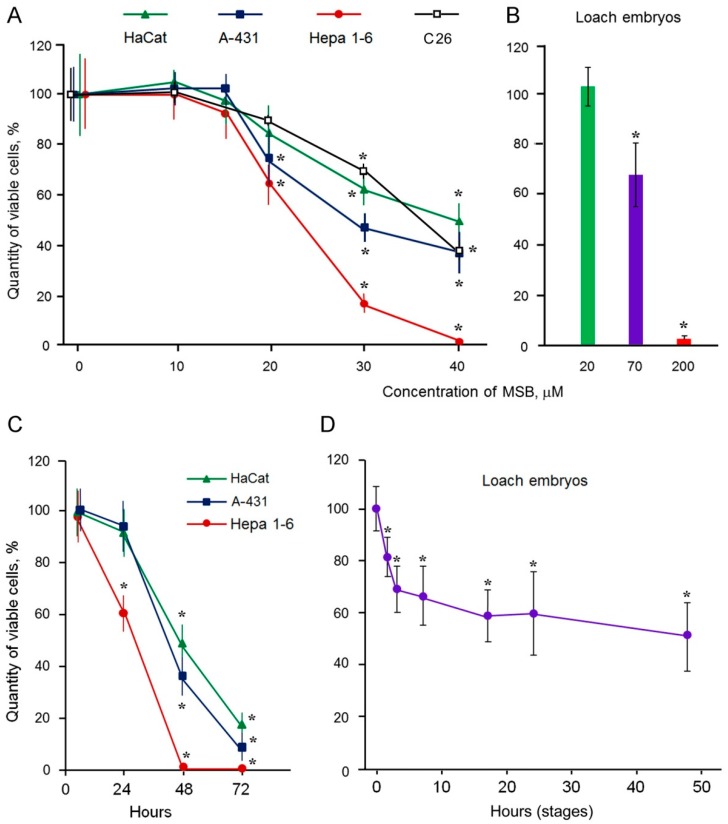
Effect of menadione sodium bisulfite (MSB) on cellular survival. Effect of different MSB concentrations (**A**,**B**); incubation during 48 h. Effect of MSB exposure duration (**C**,**D**). Incubation in the medium with 40 μM MSB (**C**). Incubation in the medium with 70 μM MSB (**D**). Standard deviation is shownn. * Significant differences from control, *p* < 0.05, *n* = 6.

MSB demonstrated the cytotoxic effect on malignant A-431 and Hepa 1-6 cells starting from 20 μM. We also detected an insignificant effect of 20 μM MSB on the survival of malignant C26 cells and immortalized HaCat cells (Figure 1A). However, 20 μM MSB was insufficient to influence the survival of loach embryos (Figure 1B), while 70 μM of MSB sufficed to induce cytotoxicity. Interestingly, temporal dynamics of MSB action differed between the cell lines and loach embryos. The cytotoxic effect of 40 μM MSB on the cell lines increased sharply after 24 to 48 h and all Hepa 1-6 cells died (Figure 1C), while the number of dead loach embryos stabilized and did not exceed 48% after 17 to 48 h of exposure to 70 μM MSB (Figure 1D).

Thus, 20 μM MSB affecting malignant cells but not normal loach embryos is suitable for antitumor composition development.

### 2.2. Suppression of Proteasome Activity by MSB In Cells

MSB is known to induce oxidative stress by the generation of superoxide radicals in the intracellular medium, which damage the tertiary and quaternary structure of proteins including proteasomes [19,21]. A cellular proteasome pool is not homogenous: cells predominantly contain 26S- and 20S-proteasomes with different mechanisms of substrate recognition [22]. 26S-proteasomes are able to recognize ubiquitinated full-size proteins and process them with the help of 19S-activator; 20S-proteasomes recognize and hydrolyze short substrates and full-size proteins with damaged structure without 19S-activator. Therefore, we were interested in the investigation of MSB influence on the total proteasome activity as well as the activities of 26S- and 20S-proteasomes.

**Figure 2:**
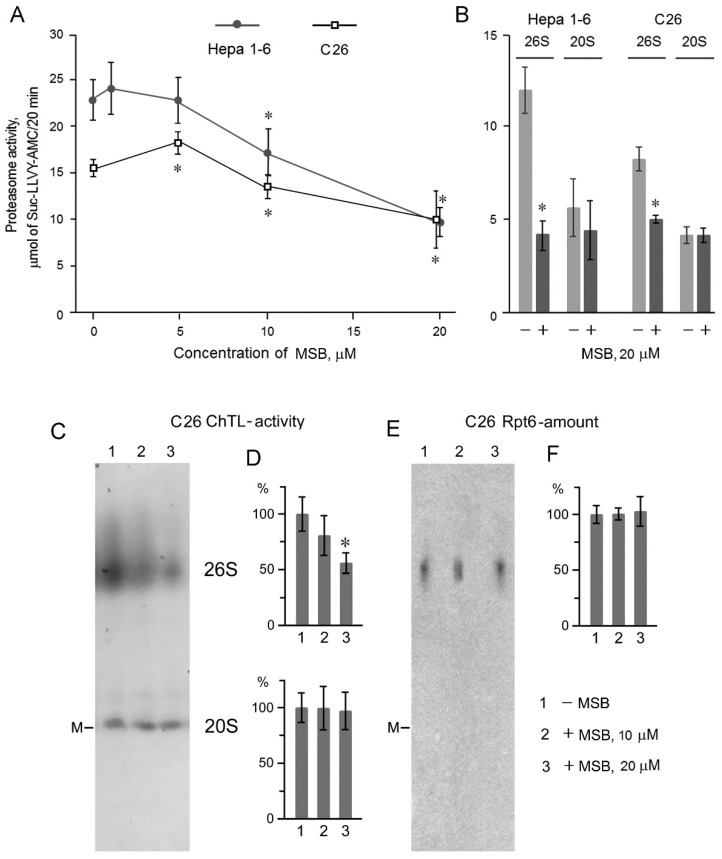
Effect of MSB on proteasome ChTL-activity in Hepa 1-6 and C26 cells. Proteasome ChTL-activity of cleared homogenates (**A**) and 26S- and 20S-proteasome fractions obtained by ammonium sulfate precipitation (**B**) and native PAGE (**C**,**D**) after incubation of cells in culture medium in the absence or presence of MSB during 48 h. Western blots of Rpt6 subunit after native PAGE (**E**) and relative Rpt6 content in the gel (**F**). ChTL-activity was normalized to 10^6^ cells (for A and B). Thyroglobulin (670 kDa), labeled by dye Cy-3.5, was used as a marker (M) of molecular mass; cleared homogenates obtained from 10^3^ cells were put on gel tracks. To identify full 20S-proteasome activity, the gel was treated with 0.04% SDS. Standard deviation is shown. * Significant differences from control, *p* < 0.05, *n* = 5.

We studied the proteasome-suppressing effects of MSB using malignant cell lines Hepa 1-6 and C26 as well as loach embryos, which have a different sensitivity to this agent (Figure 1A,B); the incubation of Hepa 1-6 and C26 cells with MSB inhibited proteasome ChTL-activity in cleared homogenates. The minimum inhibiting concentration of MSB was 10 μM (Figure 2A).

In addition, we evaluated the significant suppression of ChTL-activity of 26S-, but not 20S-proteasomes separated by ammonium sulfate precipitation after cell incubation with 20 μM MSB (Figure 2B). The sensitivity of 26S-proteasomes in tumor cells to MSB was confirmed by native gel electrophoresis (Figure 2C–F). In native 4–10% polyacrylamide gel, the total proteasome pool of C26 cleared homogenates was separated into two active forms: 26S and 20S. The incubation of C26 cells with 20 μM MSB reduced 26S-proteasome activity in the native gel by 45%, while 20S-proteasome activity did not change (Figure 2C,D). Importantly, the content of Rpt6 subunit, a marker of 19S-activator, and, hence, 26S-proteasomes was independent of MSB (Figure 2E,F). The data obtained indicate the functional damage but no quantitative reduction of 26S-proteasomes after the exposure to MSB.

**Figure 3:**
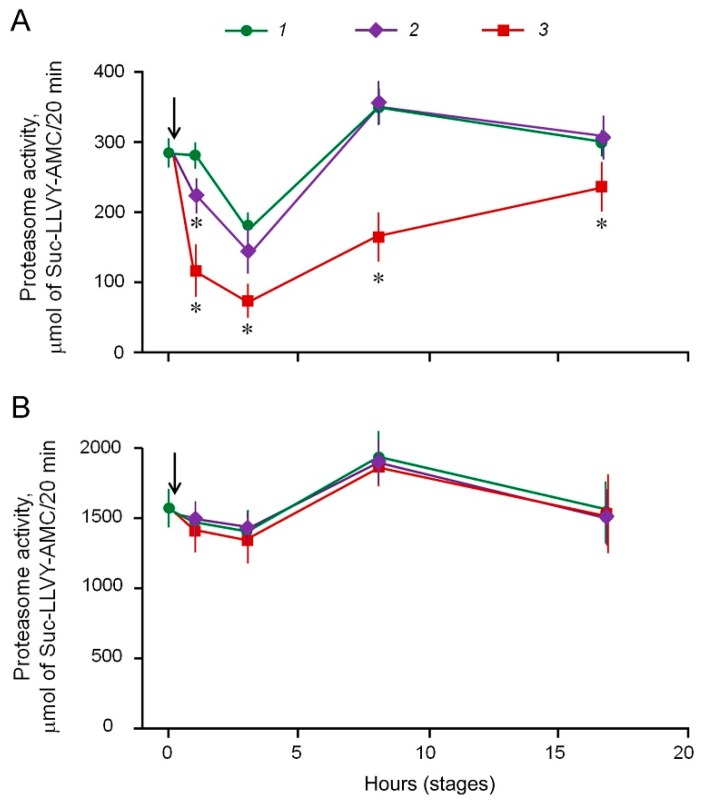
Effect of MSB exposure duration on proteasome ChTL-activity in loach embryos. Proteasome ChTL-activity of fractions enriched by 26S-proteasomes (**A**) and 20S-proteasomes (**B**) obtained by ammonium sulfate precipitation after incubation of loach embryos in the absence (1) and presence of 30 μM (2) and 70 μM MSB (3). Arrows indicate the time of MSB administration into the culture medium. ChTL-activity was normalized to 1000 embryos. Standard deviation is shown. * Significant differences from control, *p* < 0.05, *n* = 6.

At the same time, 20 μM MSB had no effect on the activity of loach embryo proteasomes. The exposure to 30 and 70 μM MSB led to the 20% and 60% suppression of 26S-proteasome ChTL-activity in loach embryos, respectively, as early as after 1 h (Figure 3A).

The effect of 30 μM MSB disappeared and the effect of 70 μM MSB became weaker in the course of embryo development. No effect of 30 and 70 μM MSB on ChTL-activity of 20S-proteasomes was observed in loach embryos (Figure 3B).

Thus, the indirect target of MSB in the proteasome pool of mammalian malignant cells and normal loach embryo cells is 26S-proteasomes containing 19S-activator, which is typical for their structure. Therefore, the suppression of 26S-proteasome activity induced by MSB is likely due to 19S-activator damage. The loach embryo model allowed us to reveal the consecutive recovery of the suppressed proteasome activity and deceleration of cell death induced by MSB in vivo. On the contrary, malignant cells were unable to overcome the damaging effects induced by MSB.

### 2.3. Content of Antitumor Compositions

**Figure 4:**
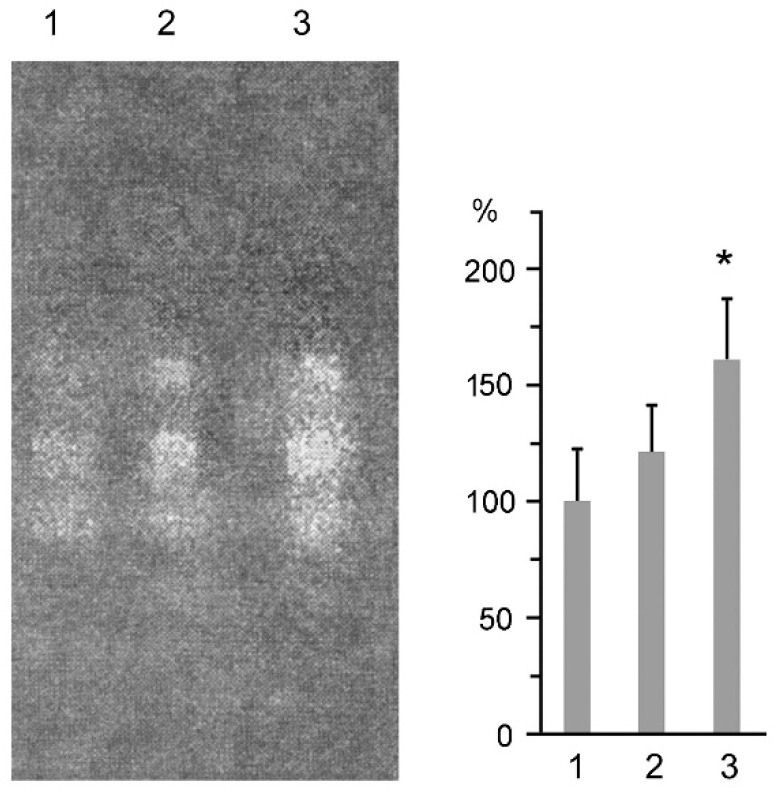
Activity of Cu, Zn-superoxide dismutase of loach embryos after their incubation in the absence or presence of MSB during 14 h. Control (1); incubation in the presence of 10 μM (2) and 20 μM MSB (3). The activity was normalized to 10^3^ embryos. Standard deviation is shown. * Significant difference from control, *p* < 0.05, *n* = 5.

On the basis of the results obtained, we chose 20 μM (6.6 μg/mL) and 10 μM (3.3 μg/mL) MSB for the development of antitumor compositions. These MSB concentrations were effective mainly against malignant cells and their proteasomes rather than against normal cells. Moreover, the activity of Cu, Zn-superoxide dismutase, known to be induced under oxidative stress to eliminate superoxide radicals [23], increased in loach embryos in the presence of 20 μM MSB (Figure 4). This fact explains the lack of toxicity of these MSB concentrations. The MSB therapeutic dose was calculated assuming that the patient blood volume is about 5 L.

Because of the high toxicity of bortezomib, we decided to diminish sharply its quantities for antitumor compositions in comparison with its current clinical dose. Therefore, we chose the bortezomib dose 4 and 10 times lower than its clinical dose on theoretical ground and tested them in the present work. Mannitol was added as an excipient to reach 38.5 mg of the total composition mass (as in antitumor drug bortezomib).

Thus, we developed the compositions BM1 (Bortezomib, MSB) containing 0.9 mg bortezomib, 33 mg MSB, 4.6 mg mannitol, and BM2 containing 0.35 mg bortezomib, 16.5 mg MSB, 21.65 mg mannitol. For reference, current antitumor drugs contain 3.5 mg bortezomib and 35 mg mannitol.

### 2.4. Effects of Bortezomib and MSB In Vitro and In Cellulo

We studied separate and mutual proteasome-inhibiting effects in vitro and the cytotoxic effect in cellulo of bortezomib and MSB applied at different concentrations including those corresponding to their amounts in BM1 and BM2.

**Figure 5:**
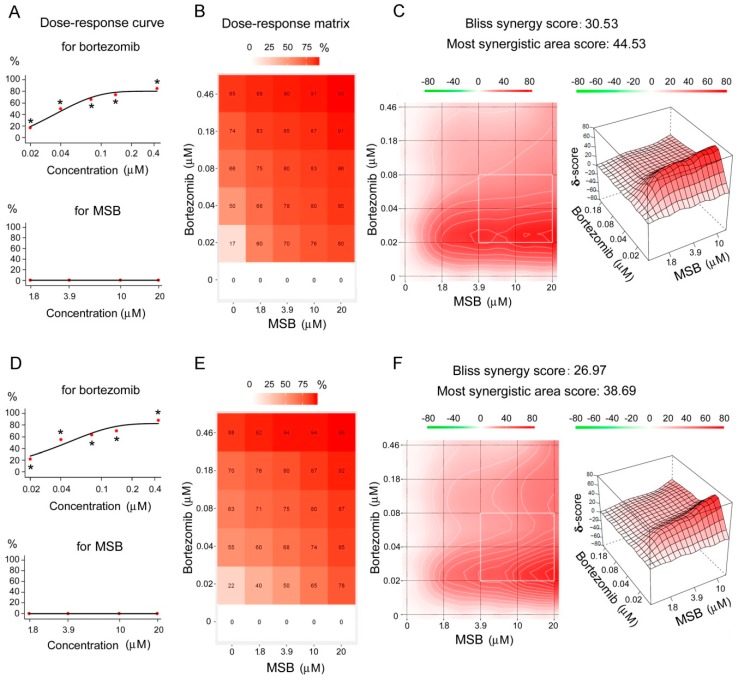
Synergetic proteasome-inhibiting effect of bortezomib and MSB in vitro. Inhibition of proteasome ChTL-activity of Hepa 1-6 cells (**A**–**C**) and A-431 cells (**D**–**F**). Dose-response curves for single chemicals (**A**,**D**). Dose-response matrix for combined chemicals (**B**,**E**). 2D and 3D synergy maps in Bliss model (**C**,**F**). Incubation of cleared homogenates with chemicals and/or proteasome substrate, 20 min, 37 °C. * Significant differences from control (in chemical absence), *p* < 0.05, *n* = 10.

Bortezomib inhibited proteasome ChTL-activity of Hepa 1-6 and A-431 cells in vitro in a concentration-dependent manner (Figure 5A,D). MSB at 1.8–20 μM did not influence proteasome ChTL-activity of these cells in vitro. Unexpectedly, these (inactive) MSB concentrations together with bortezomib showed a synergetic proteasome-inhibiting effect (Figure 5B,C,E,F), calculated using the Bliss independence model [24].

**Figure 6:**
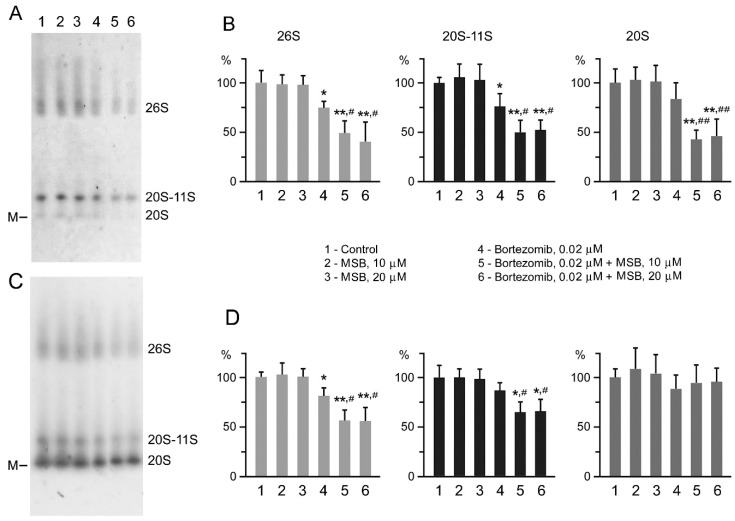
Effect of MSB and bortezomib on ChTL-activity of Hepa 1-6 cell proteasome forms in vitro. (**A**,**C**) Proteasome ChTL-activity in native gel after incubation of cleared homogenates in the absence (control) or presence of chemicals during 60 min, 37 °C. (**B**,**D**) ChTL-activity of proteasome forms in percentages with regard to the control. Proteasome activity was detected in the absence (**A**,**B**) and presence of 0.04% SDS (**C**,**D**). Thyroglobulin (670 kDa), labeled by dye Cy-3.5, was used as a marker (M) of molecular mass; cleared homogenates obtained from 10^3^ cells were put on gel tracks. Standard deviation is shown. Significant differences from control probes at *p* < 0.05 are labeled with an asterisk (*) and *p* < 0.01 with double asterisk (**), from probes in the presence of bortezomib at *p* < 0.05 with hash (#) and *p* < 0.01 with double hash (##), *n* = 5.

According to native gel electrophoresis method, MSB and bortezomib mutually influenced all proteasome forms in vitro. In particular, cleared homogenates of Hepa 1-6 cells contained three proteasome forms (Figure 6A,C): 26S, 20S, and the third form whose position coincided with that of 20S-proteasome bound to 11S-activator (PA28-activator) [25]. This form utilizes rather short substrates, generates regulatory peptides and functions as a regulatory factor in the cell [1]. Obviously, the presence of 20S-11S form is important for hepatocellular carcinoma progression. MSB had no effect on ChTL-activity of any proteasome form in the hepatocellular carcinoma in vitro, but it intensified the inhibition of all proteasome forms by bortezomib (Figure 6A,B).

We suggested that the synergetic effect of MSB and bortezomib on proteasome activity may be related to the process of bortezomib penetration into proteasome proteolytic chamber. The hydrophobic part of the MSB molecule is likely to facilitate the entry of bortezomib into proteasome proteolytic chamber by acting on the hydrophobic “stopper” of proteasome α-subunits. In this case, MSB action is not enough to facilitate the entry of a larger substrate molecule into proteasome. To clarify this suggestion, we further analyzed the activity of each proteasome form in the presence of a 0.04% SDS opening the entry into a 20S-proteasome channel completely [26]. In these conditions, MSB increased the inhibition of 26S and 20S-11S forms by bortezomib, however, this effect was not revealed for the 20S form (Figure 6C,D). Note, 20S-proteasome activity increased significantly in the presence of 0.04% SDS (Figure 6A,C). Obviously, bortezomib was displaced by the substrate, which led to the disappearance of the mutual effect of MSB and bortezomib on 20S-proteasomes. This result confirmed that the effect of MSB on the inhibition of substrate hydrolysis by bortezomib was associated with the process of substance penetration into the proteolytic chamber.

**Figure 7:**
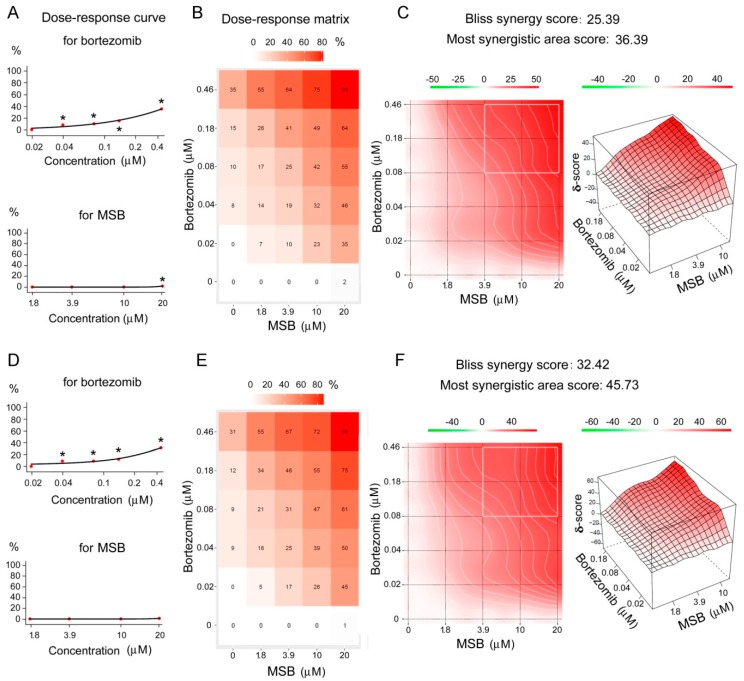
Synergetic cytotoxic effect of bortezomib and MSB. Cytotoxic effect against Hepa 1-6 cells (**A**–**C**) and A-431 cells (**D**–**F**). Dose-response curves for single chemicals (**A**,**D**). Dose-response matrixes for combined chemicals (**B**,**E**). 2D and 3D synergy maps in Bliss model (**C**,**F**). Cells were incubated in the presence of chemicals for 24 h * Significant differences from control (in chemical absence), *p* < 0.05, *n* = 7.

In addition, bortezomib showed a cytotoxic effect against Hepa 1-6 and A-431 cells dependent on its concentration. MSB displayed a very low cytotoxic activity at 20 μM against Hepa 1-6 cells (Figure 7A,D). MSB together with bortezomib showed a mutual synergetic cytotoxic effect (Figure 7B,C,E,F) calculated by the Bliss method.

**Figure 8:**
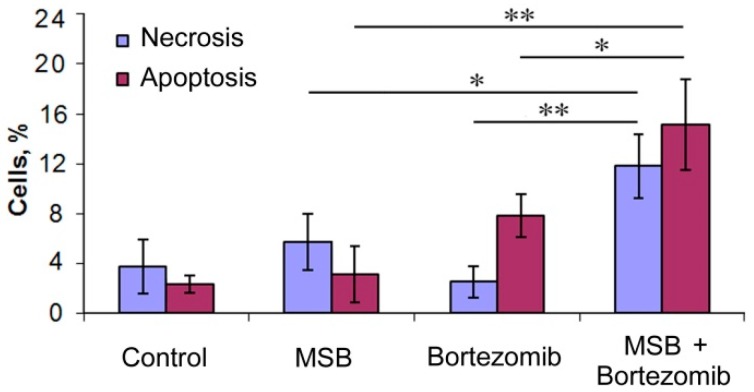
Number of necrotic and apoptotic Hepa 1-6 cells after their incubation with MSB (10 μM) and/or bortezomib (0.04 μM). Data are presented as the mean ± standard deviation. * *p* < 0.05, ** *p* < 0.01, *n* = 4.

Note, bortezomib and MSB induced both necrosis and apoptosis of tumor cells in a synergetic manner (Figure 8).

The strongest synergetic proteasome-inhibiting effect was observed for low bortezomib concentrations (0.02–0.04 μM) combined with all studied concentrations of MSB (Figure 5C,F). However, the most considerable synergetic cytotoxic effect was revealed for high bortezomib doses (0.08–0.46 μM) (Figure 7C,F). Similar results were obtained with the use of the HSA (Highest Single Agent) (Figure S1) and ZIP (Zero Interaction Potency) models (Figure S2). These results may be explained by the difference between dose-response curves of the most active component, bortezomib, for proteasome inhibition and cytotoxicity. Obviously, the synergetic effect is more pronounced at the background of 20–50% (rather than 70–80%) inhibition of proteasome activity (Figure 5A,C,D,F) as well as on the background of 15–40% cytotoxic process (Figure 7A,C,D,F).

The data obtained indicates that the components of BM1 and BM2 have a combined effect on proteasomes upon entering the cell. First, MSB causes an indirect functional damage of 26S-proteasomes mainly in tumor cells. This effect relies on the capability of the quinone structure of the MSB molecule to generate superoxide radicals in the intracellular medium. Second, bortezomib and MSB cause a synergetic inhibition of ChTL-activity of the total proteasome pool. Possibly, in this case, the hydrophobic part of the MSB molecule facilitates the entry of bortezomib into the proteasome proteolytic chamber by acting on the hydrophobic “stopper” formed by the proteasome α-subunits. Indicated events lead to the synergetic cytotoxic effect mainly against tumor cells. These facts together allowed us to suggest the high antitumor efficiency and low toxicity of the developed compositions in vivo. We tested this suggestion in a preclinical trial on mouse and rat models.

### 2.5. Toxicity of BM1 and BM2 In Vivo

**Figure 9:**
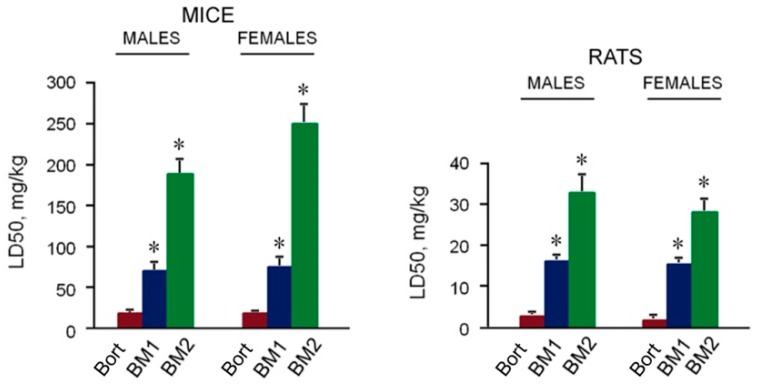
LD50 of the developed compositions and bortezomib. Data are presented as the mean ± standard deviation. * Difference from bortezomib at *p* < 0.001, *n* = 20. Bort, Bortezomib.

The toxicity of BM1 and BM2 was evaluated by semi-lethal dose (LD50) for white outbred mice and white outbred rats in comparison with bortezomib. LD50 magnitudes of BM1, BM2 and bortezomib were considerably higher for mice compared to rats (Figure 9). For male and female mice, LD50 of BM1 was 4 times higher than LD50 of bortezomib, and LD50 of BM2 was 10 times higher for males and 13 times higher for female mice than LD50 of bortezomib. LD50 of BM1 was 6 times higher for male and 7 times higher for female rats than LD50 of bortezomib, LD50 of BM2 was 12 times higher for male and 14 times higher for female rats than LD50 of bortezomib. On the whole, the toxicity of BM1 and BM2 was 4–7 and 10–14 times lower, respectively, than bortezomib with regard to tested rodents.

Note, the sensitivity of male and female mice to BM2 was different. Obviously, male, and female mice were able to reduce the effects of BM2 in a different manner depending on their hormonal status. As a result, female mice were more resistant to BM2: LD50 for them was higher by 25%. However, neither male nor female mice were able to reduce successfully the negative effects of more toxic BM1 and bortezomib. Therefore, LD50 magnitudes of BM1 or bortezomib for male and female mice did not differ. BM2, BM1, and bortezomib were much more toxic for rats than for mice regardless of the sex. There was no reliable difference (*p* > 0.05) in LD50 magnitudes for male and female rats. Thus, the difference in response of male and female rodents to drugs depends on the degree of drug toxicity.

### 2.6. Antitumor Effects of BM1 and BM2 In Vivo

Before the investigation of the antitumor effects of BM1, BM2, and bortezomib, the maximum tolerated dose (MTD) was estimated for every composition. The MTDs of BM1, BM2, and bortezomib were 64.1, 164.8, and 16.5 mg/kg, respectively, for males and females of C57Bl/6 mice. The indicated doses did not cause the death of the animals during the observation period. During this period, the loss of animal body mass did not exceed 10%. However, the indicated doses provoked the death of C57Bl/6 mice with grafted Hepa 1-6 cells. For these mice, the MTDs of BM1, BM2, and bortezomib were 21.4, 55.0, and 5.5 mg/kg, respectively.

**Figure 10:**
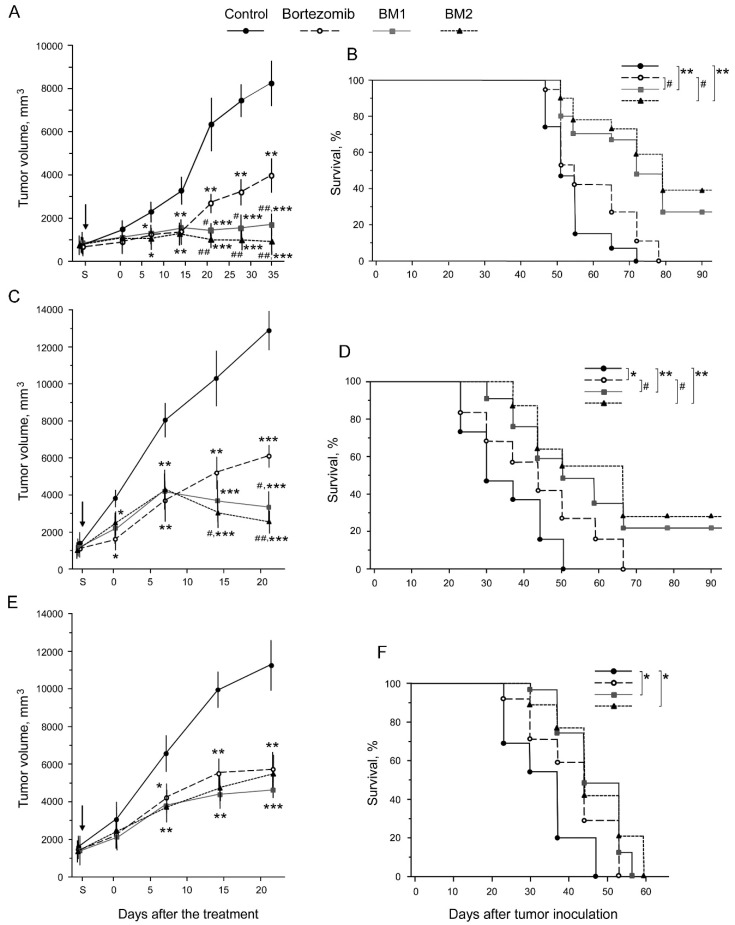
Antitumor efficiency of the developed compositions and bortezomib in vivo. Data for hepatocellular carcinoma Hepa 1-6 (**A**,**B**), mammary adenocarcinoma Ca 755 (**C**,**D**), and Lewis lung carcinoma LLC1 (**E**,**F**). Tumor growth in C57Bl/6 mice (**A**,**C**,**E**) and Kaplan–Meier survival curves (**B**,**D**,**F**) in the absence or presence of compositions. Arrows show the start of the treatment (S). Difference from control at *p* < 0.05 is labeled with an asterisk (*), *p* < 0.01 with a double asterisk (**), *p* < 0.005 with a triple asterisk (***), from bortezomib at *p* < 0.05 with hash (#), *p* < 0.01 with double hash (##); *n* = 50 in total for every animal group at the beginning of investigation (10 animals × 5 experiments).

To assess the antitumor effects of the developed compositions in vivo, three models of socially significant solid tumors were used: hepatocellular carcinoma Hepa 1-6, mammary adenocarcinoma Ca 755, and Lewis lung carcinoma LLC1, which were grafted into C57Bl/6 mice (Figure 10). For the comparison of the antitumor effects of the developed compositions and bortezomib, the same dose of 5.5 mg/kg not exceeding the MTD of bortezomib (the most toxic of them) was used for all investigated compositions. All compositions suppressed tumor growth (Figure 10A,C,E). Importantly, BM1 and BM2 suppressed the growth of hepatocellular carcinoma and mammary adenocarcinoma more effectively than bortezomib. Moreover, by the 35th day after the last injection of BM2, the volume of hepatocellular carcinoma was close to the initial volume of this tumor, while after the treatment by bortezomib the tumor was growing (Figure 10A). Besides that, BM1 and BM2 increased the survival of mice with any of the grafted tumors compared to the control (Figure 10B,D,F). The increase in the survival of mice with grafted hepatocellular carcinoma or mammary adenocarcinoma after the treatment by BM1 and BM2 was more significant than that of bortezomib (Figure 10B,D). Antitumor effects of BM1 and BM2 against lung carcinoma did not differ from the effect of bortezomib (Figure 10F).

It is very important to note that in the mice who survived after the treatment by BM1 and BM2, hepatocellular carcinoma and mammary adenocarcinoma were not detected during the entire period of observation; 150 days after the inoculation.

Thus, we developed two unique antitumor compositions, BM1 and BM2, which displayed much lower toxicity, by 4–14 times, for rodents in comparison with bortezomib. In the background of such low toxicity, both compositions showed antitumor effects either higher than bortezomib (for hepatocellular carcinoma and mammary adenocarcinoma) or similar to bortezomib (for Lewis lung carcinoma). If we compare BM1 and BM2 compositions and try to make a choice between them, we can argue for BM2. On the one hand, the toxicity of BM2 was dramatically lower, by 10–14 times, than the toxicity of bortezomib and 2–3 times lower than the toxicity of BM1. On the other hand, the antitumor efficiency of BM2 was not less than that of BM1.

Thus, the developed antitumor compositions are advantageous compared to single MSB or bortezomib. This may be explained by the higher cytotoxicity of the compositions against tumor cells compared to single MSB applied at the doses like in the compositions. Larger doses of MSB as a single antitumor chemical are not reasonable to use due to their ability to cause serious oxidative stress in the body. Note, the MSB doses in BM1 and BM2 do not exceed the maximum daily dose of this chemical in current therapy of prothrombinopenia. Much lower acute toxicity and comparable or even higher antitumor efficiency of the developed compositions in relation to bortezomib ensured the advantage of the compositions compared to a single bortezomib. The low acute toxicity of BM1 and BM2 is related to the low bortezomib doses in them and, perhaps, to the difference in the ratio and functions of the proteasome forms in the tumor and normal mammalian cells.

**Figure 11:**
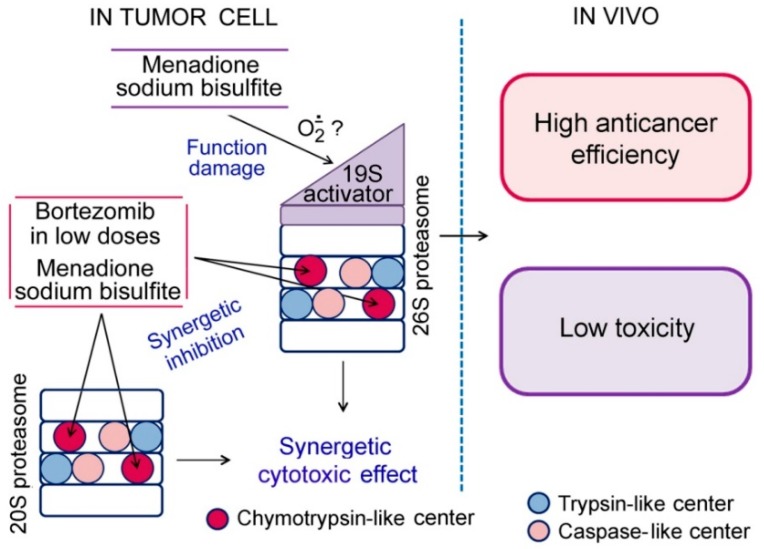
The schematics presenting the mechanisms of action of the developed compositions.

At the same time, the present work has the limitation related to the study of only two combinations of bortezomib and MSB and only three tumor types in vivo. Nevertheless, the results obtained so far are sufficient to assert the connection of the combined effects of the developed compositions on proteasomes in tumor cells with the high antitumor activity and low toxicity of these compositions in vivo (Figure 11). Further development of compositions based on combinations of bortezomib and MSB promises the development of novel effective antitumor drugs which may outperform the current proteasome-inhibiting drugs bortezomib, carfilzomib, and ixazomib [11,14] in a very low general toxicity.

Application of another proteasome inhibitor BSc2118 in mice induced local effects against melanoma [27]. Systemic administration of BSc2118 in mice was tolerated at higher doses as compared to bortezomib. However, antitumor effects and toxicity of BM1 and BM2 compositions have been studied in more detail. Since the components of BM1 and BM2, bortezomib and MSB, are used in medical practice now, the development and registration of novel drugs based on these chemicals may be facilitated.

## 3. Materials and Methods

### 3.1. Animals and Cell Lines

C57Bl/6 mice (840 animals), white outbred mice (120 animals) and white outbred rats (120 animals) were purchased from the Stolbovaya Nursery (Russian Academy of Sciences). Animals were acclimated to standard conditions for two weeks. Mice of 18–22 g and rats of 176–189 g were used. Experiments were carried out in accordance with the European Communities Council Directive of 24 November 1986 (86/609/EEC). All the protocols of manipulations with animals have been approved by the Commission on Bioethics of Koltzov Institute of Developmental Biology, Russian Academy of Sciences (Permit Number: 12/2.4). Embryos of teleost fish *Misgurnus fossilis* (loach) were obtained as described earlier [28].

Human epidermoid carcinoma cells A-431 and immortalized keratinocytes HaCat were obtained from Cell Culture Collection of Koltzov Institute of Developmental Biology, Russian Academy of Sciences. Murine Lewis lung carcinoma and mammary adenocarcinoma Ca 755 were obtained from Blokhin Russian Cancer Research Center. Murine hepatocellular carcinoma Hepa 1-6 was obtained from Engelhardt Institute of Molecular Biology, Russian Academy of Sciences, murine colon adenocarcinoma C26 was obtained from Hertsen Moscow Oncology Research Center, Department of Anticancer Therapy Modifiers and Protectors.

### 3.2. Chemicals

MSB (3-dihydro-2-methyl-1,4-naphthoquinone-2-sulfonate sodium; C_11_H_9_NaO_5_S·3H_2_0) (Close Corporation NIKKA, Moscow, Russia) and bortezomib ([(1R)-3-Methyl-1-[[(2S)-1-oxo-3-phenyl-2-[(pyrazinylcarbonyl)amino]propyl]amino]butyl] boronic acid; C_19_H_25_BN_4_O_4_) (Veropharm, Moscow, Russia) were used. For a preclinical trial on rodent models, pharmaceutical medicine bortezomib (Velcade) (Pierre Fabre Medicament Production, France) was used. Compositions of MSB, bortezomib and mannitol (as an excipient) were prepared in Mendeleev University of Chemical Technology of Russia.

### 3.3. Treatment of Cells with MSB

Cells were incubated in Dulbecco’s modified Eagle medium supplemented with 10% heat-inactivated fetal calf serum (Hyclone, Logan, UT, USA), 2 mM L-glutamine and 50 IU/mL penicillin and 50 μg/mL streptomycin (PanEco, Moscow, Russia) in the absence (control) or presence of MSB (1–40 μM) at 37 °C, 5% CO_2_, and 95% humidity for 24–72 h.

Loach eggs obtained by stimulation of fish by gonadotropin were fertilized in Petri dishes [18]; 5 min after fertilized eggs were incubated in sterile pond water in the presence of MSB (20–200 μM) at 21.5 °C for 1–48 h. Intact embryos served as a control.

### 3.4. Preparation of Proteasome Fractions

Cell culture medium containing floating dead cells was removed. Attached surviving cells were washed with PBS for further proteasome fraction preparation. Dead loach embryos were easily visualized by their white color and loss of shell transparency. Fertilized eggs and surviving embryos at different development stages were collected with a pipette.

Cleared homogenates of cells and embryos were prepared as described previously [6]. Fractions enriched in 26S- and 20S-proteasomes were obtained by ammonium sulfate precipitation (0–40% and 40–70% fractions, respectively) [26]. In addition, proteasome pool of tumor cell cleared homogenates was divided into 26S- and 20S-proteasomes by native 4–10% PAGE developed for crude proteasome fractions [25].

### 3.5. Determination of Proteasome ChTL-Activity and Superoxide Dismutase Activity

Proteasome ChTL-activity was determined by hydrolysis of fluorogenic Suc-LLVY-AMC (Sigma-Aldrich, St. Louis, MO, USA) in 0.5, 1.0, 1.5, and 2.0 μL of the proteasome fraction as described previously [25]. ChTL-activity was expressed as the substrate quantity (μmol of Suc-LLVY-AMC) hydrolyzed at 37 °C for 20 min and normalized to 10^6^ cells or 1000 embryos. Under indicated conditions, the quantity of the reaction product is proportional to reaction duration [25].

Proteasome ChTL-activity in native 4–10% polyacrylamide gel was detected with the use of 300 μM Suc-LLVY-AMC (1/20 of gradient gel volume) as described previously [25]. Fluorescence bands in the gel were photographed under 365 nm UV light.

Cu, Zn-superoxide dismutase activity was detected in 10% polyacrylamide gel by the standard procedure involving the competition with nitroblue tetrazolium for a photochemical flux of superoxide radicals [29]. The image analysis of proteasome and superoxide dismutase activities in gels was performed using the standard ImageJ software (https://imagej.nih.gov/ij/).

### 3.6. Western Blotting

After native PAGE and semidry transfer of polypeptides onto a Hybond-ECL membrane (Amersham) [25], immunodetection was carried out by the standard method using the primary mouse monoclonal antibodies to proteasome 19S ATPase subunit Rpt6 (product number: BML-PW9265, lot number: X09256, Enzo Life Sciences, Farmingdale, NY, USA), 1:1500, and the corresponding peroxidase-conjugated secondary antibodies. The image analysis was performed using the standard ImageJ software. The relative quantities (optical density) of the immunoreactive bands on an X-ray film were measured. The dependence of the optical density on the amount of the protein subjected to Western blotting was evaluated preliminarily. For further processing, portions were taken within a proportional dose range.

### 3.7. Assay for Cytotoxic Effects

Cytotoxic effects of MSB and bortezomib on cells were evaluated with the use of the standard colorimetric assay based on the ability of viable cells to transform 3-(4,5-dimethylthiazol-2yl)-2,5-diphenyltetrazolium bromide (MTT) to purple formazan crystals soluble in organic solvents [30].

After incubation of cells in culture media with or without effectors, 0.45 mg/mL MTT was added to the probes and kept in CO_2_ incubator for 4 h at 37 °C. The culture medium was replaced with isopropanol for 5 min at room temperature. The quantity of formazan (proportional to the number of viable cells) was measured as absorbance at 550 nm. The reference wavelength was set at 620 nm. The final calculation was the subtraction of the absorbance magnitude at 620 nm from that at 550 nm. The number of viable cells in the presence of effectors was presented as a percentage of the control magnitude (without effectors).

### 3.8. Detection of Apoptosis and Necrosis

Apoptosis and necrosis were measured using FITC conjugated Annexin V and SYTOX™ Blue Dead Cell Stain (Thermo Fisher Scientific, Waltham, MA, USA), respectively, as described previously [31]. Hepa 1-6 cells were seeded into the 6 well plate (25,000 per well) and incubated overnight. Then cells were incubated in the absence or presence of 10 μM MSB and/or 0.04 μM bortezomib for 24 h. Following incubation, cells were detached using trypsin, washed with PBS and resuspended in 100 μL of Staining buffer (140 mM NaCl, 20 mM HEPES, 2.6 mM CaCl_2_). Then Annexin V-FITC solution (2 μL) was added to each sample. Samples were left for 15 min in the dark. After that, they were transferred on ice and mixed with 300 L of ice-cold Staining buffer. Finally, 1 μL (1 mg/mL) of SYTOX was added. All measurements were performed with the use of a BD LCRFontessa flow cytometer (BD Biosciences, San Jose, CA, USA).

### 3.9. Tumor Strain Maintaining

Tumor strains were maintained in female (Ca 755 cells) and male C57Bl/6 mice (LLC1 and Hepa 1-6 cells). Tumor suspension (0.3 mL of Ca 755 and Hepa 1-6 cells or 0.5 mL of LLC1 cells) prepared in culture medium 199 (1 ÷ 10, w ÷ v) was subcutaneously injected into mice for 14–20 days (Ca 755 and LLC1 cells) or 18–28 days (Hepa 1-6 cells).

### 3.10. Inoculation of Tumor Cells

Tumors that passed at least two passages in mice were used. Suspension of Ca 755 cells (0.3 mL) prepared in culture medium 199 (1 ÷ 10, w ÷ v) was subcutaneously injected into female C57Bl/6 mice. Suspension of LLC1 cells (0.5 mL) prepared in culture medium 199 (1 ÷ 10, w ÷ v) was subcutaneously injected into male C57Bl/6 mice. The treatment of mice by the compositions started 48 h after the tumor inoculation.

Suspension of Hepa 1-6 cells (0.1 mL) prepared in culture medium 199 (1 ÷ 5, w ÷ v) was subcutaneously injected into male and female C57Bl/6 mice. The treatment of mice by the compositions started after the tumor size reached not less than 400 mm^3^.

### 3.11. Evaluation of Antitumor Effects

The bortezomib-MSB compositions or bortezomib in 0.9% NaCl were intraperitoneally administered 6 times (twice a week) into C57Bl/6 mice with grafted tumors in doses depending on the body mass and not exceeding MTD; saline was given to control animals. The tumor volume was evaluated by the probit method and expressed in mm^3^. Dead animals were counted for 150 days after tumor inoculation.

### 3.12. MTD Detection

For the detection of MTDs, the bortezomib-MSB compositions or bortezomib in saline were intraperitoneally administered 6 times (twice a week) into intact C57Bl/6 mice and C57Bl/6 mice with grafted Hepa 1-6 cells. After the last administration, the animals were followed for three weeks. During this period, the general condition of animals was evaluated by their motor activity, food and water intake, the condition of the fur, and body mass. Saline was used for the control animals.

### 3.13. Evaluation of LD50

For evaluation of LD50, the bortezomib-MSB compositions and bortezomib in saline were injected once into the caudal vein of white outbred mice and white outbred rats in the doses depending on the animal mass. Dead animals were counted within 24 h after the administration of compositions.

### 3.14. Statistics

Differences from the control values were assessed using the Kruskal–Wallis test of ANOVA and 95% confidence intervals. Significant differences were considered at *p* < 0.05. The variances of the data were evaluated with the F-test. The statistical analysis was performed by Statistica 8.0 (Statsoft, 2008, https://www.tibco.com/products/tibco-statistica). Kaplan–Meier survival data were analyzed by the log-rank test.

## 4. Conclusions

We have developed anticancer compositions containing bortezomib in amounts 4 and 10 times lower than its current therapeutic dose and MSB in amounts effective against tumor cells (but not normal cells) and their proteasomes.

The components of the developed compositions showed a combined action on proteasomes of tumor cells: MSB indirectly suppressed 26S-proteasome function, and MSB together with bortezomib inhibited ChTL-activity of the total proteasome pool in a synergetic manner. These influences led to the synergetic cytotoxic effect.

The discovered characteristics of bortezomib and MSB are closely related to the properties of the developed compositions revealed in rodent models in the preclinical trial. First, both compositions displayed much lower (4–14 times) acute toxicity compared to bortezomib. Second, in the background of low toxicity, both compositions showed antitumor effects either higher (for hepatocellular carcinoma and mammary adenocarcinoma) or similar to bortezomib (for Lewis lung carcinoma).

Thus, the developed compositions represent the novel generation of bortezomib-based anticancer drugs combining high efficiency, low general toxicity, and a potentially expanded set of target tumors.

## 5. Patents

Sharova, N.P.; Astakhova, T.M.; Morozov, A.V.; Erokhov, P.A.; Lyupina, Yu.V.; Mikhailovskaya, M.I., Chupikova, N.I.; Safarov, R.R. Synergetic combination of proteasome inhibitor and vitamin K for the inhibition of tumor cell growth and proliferation, pharmaceutical composition, and antitumor drug on its basis. RU 2563986. 2015. (Patent holder: N.K. Koltsov Institute of Developmental Biology of Russian Academy of Sciences).

## Supplementary Materials

The following are available online at http://www.mdpi.com/2072-6694/10/10/351/s1, Figure S1: 2D and 3D synergy maps in HSA model for bortezomib and MSB effects. Inhibition of proteasome ChTL-activity of Hepa 1-6 cells (A) and A-431 cells in vitro (B). Cytotoxic effects against Hepa 1-6 cells (C) and A-431 cells (D), Figure S2: 2D and 3D synergy maps in ZIP model for bortezomib and MSB effects. Inhibition of proteasome ChTL-activity of Hepa 1-6 cells (A) and A-431 cells in vitro (B). Cytotoxic effects against Hepa 1-6 cells (C) and A-431 cells (D).
