# Supplementary material for: Combined Effect of Bortezomib and Menadione Sodium Bisulfite on Proteasomes of Tumor Cells: The Dramatic Decrease of Bortezomib Toxicity in a Preclinical Trial
Source: Cancers (Basel). 2018 Sep 25;10(10):351. doi: 10.3390/cancers10100351 (PMC6209890; doi:10.3390/cancers10100351)
Supplement: Supplementary file 1 [file cancers-10-00351-s001.pdf]

Supplementary Materials: Combined Effect of Bortezomib and Menadione Sodium Bisulfite on Proteasomes of Tumor Cells: The Dramatic Decrease of Bortezomib Toxicity in a Preclinical Trial

Tatiana M. Astakhova, Alexey V. Morozov, Pavel A. Erokhov, Maria I. Mikhailovskaya, Sergey B. Akopov, Natalia I. Chupikova, Ruslan R. Safarov and Natalia P. Sharova

Supplementary Figures

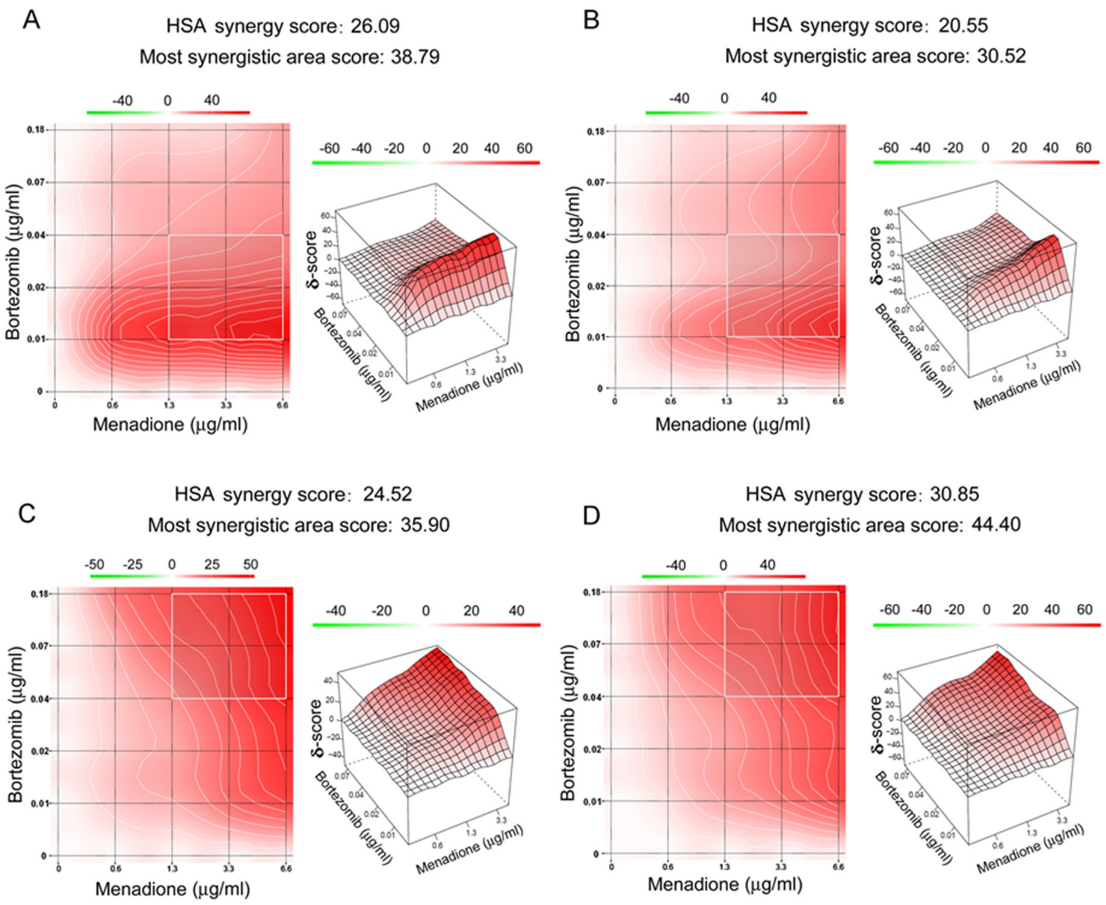

Figure S1. 2D and 3D synergy maps in HSA model for bortezomib and menadione effects. Inhibition of proteasome ChTL-activity of Hepa 1-6 cells (**A**) and A-431 cells in vitro (**B**). Cytotoxic effects against Hepa 1-6 cells (**C**) and A-431 cells (**D**).

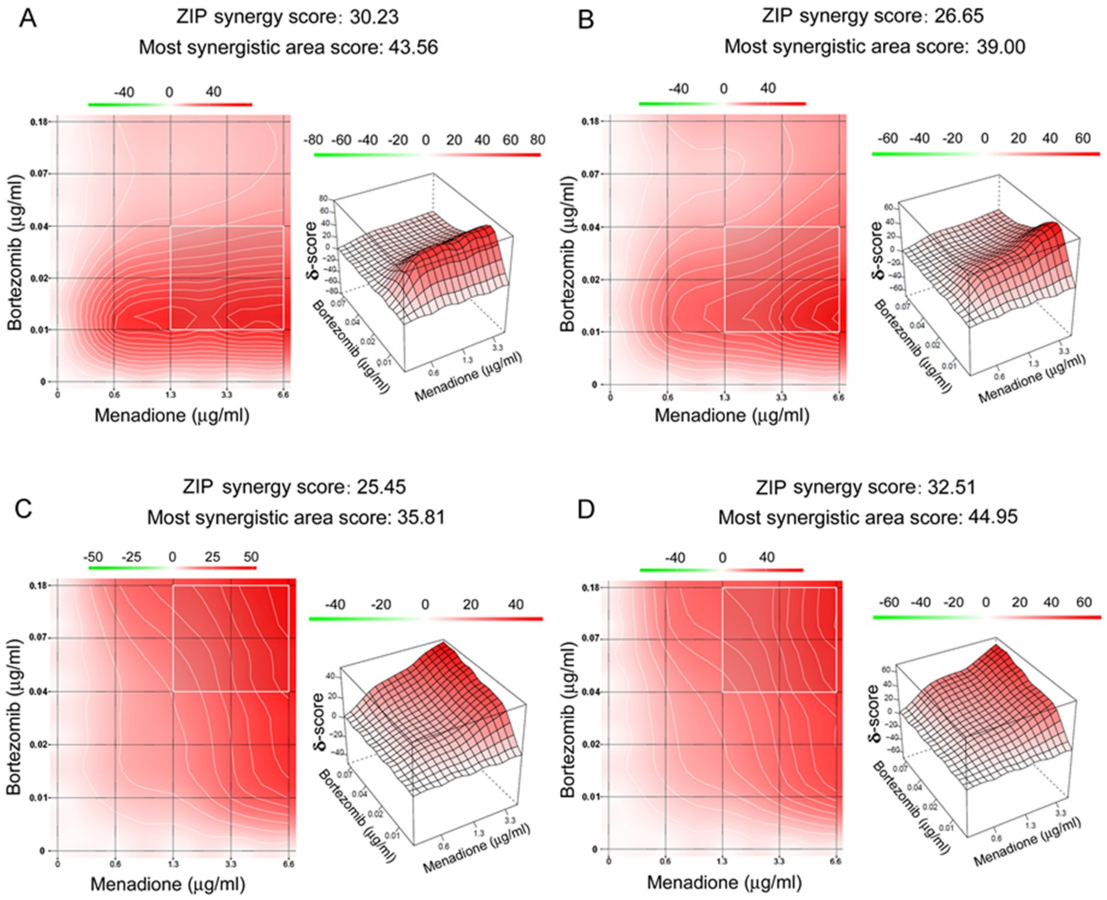

Figure S2. 2D and 3D synergy maps in ZIP model for bortezomib and menadione effects. Inhibition of proteasome ChTL-activity of Hepa 1-6 cells (**A**) and A-431 cells in vitro (**B**). Cytotoxic effects against Hepa 1-6 cells (**C**) and A-431 cells (**D**).

© 2018 by the authors. Licensee MDPI, Basel, Switzerland. This article is an open access article distributed under the terms and conditions of the Creative Commons Attribution (CC BY) license (http://creativecommons.org/licenses/by/4.0/).
